# A Multidisciplinary Approach to the Management of Atypical Osseous Epithelioid Hemangioendothelioma

**DOI:** 10.1155/2014/917425

**Published:** 2014-02-18

**Authors:** J. K. Ma, J. Barr, S. Vijayakumar

**Affiliations:** ^1^Department of Radiation Oncology, University of Mississippi Medical Center, 350 Woodrow Wilson Drive, Suite 1600, Jackson, MS 39213, USA; ^2^Department of Orthopedics Surgery, University of Mississippi Medical Center, 2500 North State Street, Jackson, MS 39216, USA

## Abstract

Hemangioendothelioma is a rare vascular tumor of intermediate malignant potential. Though epithelioid hemangioendothelioma (EH) is commonly found in soft tissues, it has been known to be present in skeletal tissues. The authors present a case of a 50-year-old woman diagnosed with EH of the iliac bone and acetabulum, who experienced pathological fracture at presentation. This report describes a multidisciplinary approach to the management that includes initial incisional biopsy, curettage, and bone grafting, followed by Intensity Modulated Radiation Therapy. The patient finally underwent hemipelvic resection with allograft reconstruction after recurrence. Histopathological study revealed osseous EH of low mitotic activity that stained positively for CD31, CD34, vimentin, and Factor VIII. Herein, the authors discuss the imaging characteristics, histopathological aspects, cytogenetic findings, and the radiobiological behavior of osseous EH. After an aggressive multidisciplinary intervention, the patient is able to achieve local control with no evidence of distal metastatic disease.

## 1. Introduction

Hemangioendothelioma is a rare tumor that exhibits intermediate malignant potential, clinically behaving between benign hemangioma and malignant angiosarcoma. Epithelioid hemangioendothelioma (EH) is the most common histologic subtype that arises from vascular tissue [1] and represents less than one percent of all vascular neoplasms [2]. EH is most commonly found in soft tissues but can also be found in skeletal tissues such as skull, spine, pelvis, femur, and tibia [2]. Primary bone EH accounts for less than one percent of all malignant bone tumors [3]. For certain isolated tumors, curative resection with negative margins can achieve cure and long term local control. Role of chemotherapy and adjuvant radiation therapy remains unclear. Here, we report and discuss the management of epithelioid hemangioendothelioma of the acetabulum and the ilium in the setting of multidisciplinary approach, including orthopedic surgery, radiation oncology, medical oncology, interventional radiology, and pathology.

## 2. Case Report

### 2.1. Clinical History

**Figure 1:**
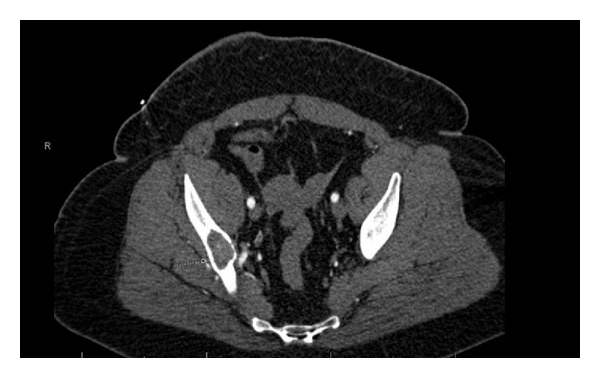
Preoperative IV contrast enhanced axial CT showing the enhancing mass in the right iliac bone.

50-year-old African American woman initially presented to the emergency department with a two-week history of vague right hip and lower pelvic pain. Her past medical history was significant for rheumatoid arthritis, diabetes mellitus type 1, and hyperthyroidism status after radioactive iodine ablation. No history of trauma or systemic etiology was found. Physical exam was unremarkable for motor or sensory neurological deficits. No functional impairment was detected. CT of abdomen and pelvis obtained at an outside facility revealed an enhancing mass identified within the medullary space of the right iliac bone which extends from the level of sacroiliac joint to the roof of right acetabulum (Figure 1). Patient first underwent CT guided biopsy of the right acetabular mass. A pathological fracture was identified within the medial wall of the right iliac bone. Patient underwent incisional biopsy, curettage, and bone graft of right posterior wall of acetabulum. Intraoperatively, a large cavitary mass defect of ischium was noted to involve the posterior wall of acetabulum up to greater sciatic notch. In the operating room, the rim of the posterior wall of the acetabulum was outlined. The roof of the expansile bony lesion was fenestrated and unroofed. The cystic content beneath, which consisted of soft globular solid pink material, was curettaged.

**Figure 2:**
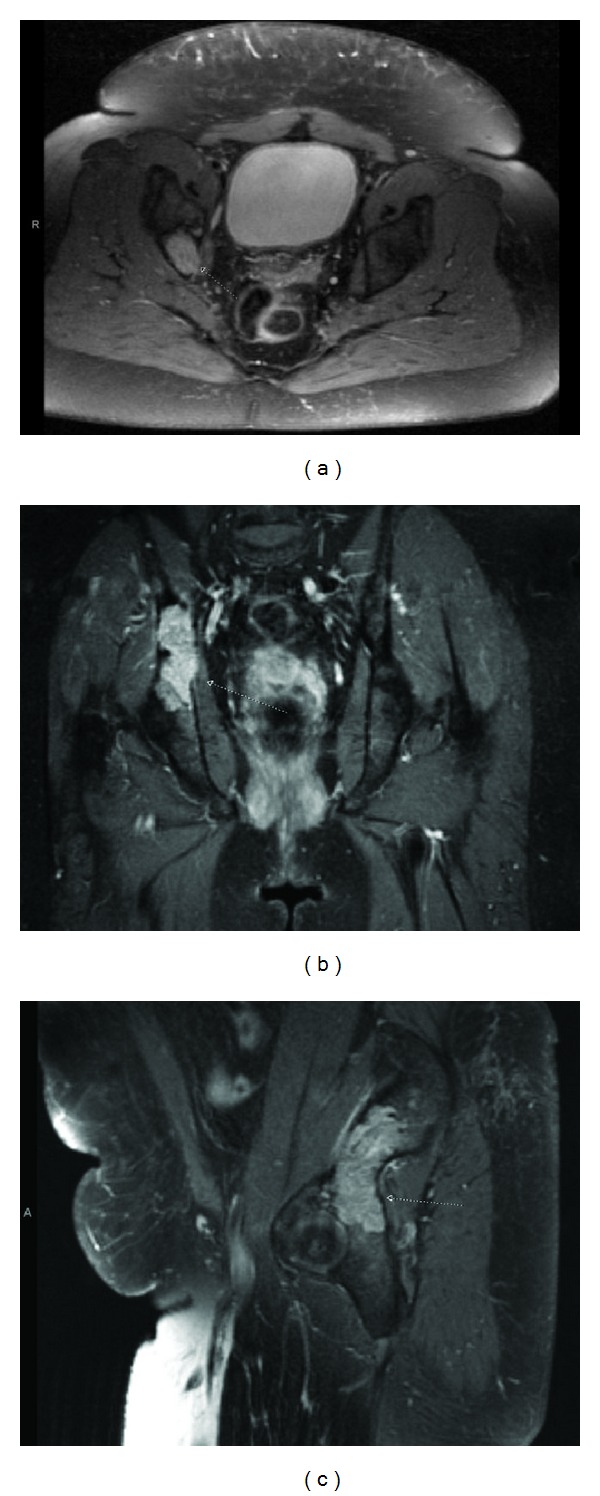
Preradiotherapy axial (a), coronal (b), and sagittal (c) postcontrast T1-weighted magnetic resonance imaging scans showing a right iliac heterogeneously enhancing mass.

Postoperative MRI of the pelvis (Figure 2) noted minimal edema in the posterior gluteus maximus and medius fibers. The residual right iliac mass demonstrated cortical thinning and was homogeneously isointense to muscle on T1-weighted sequences and mildly heterogeneous but hyperintense relative to adjacent muscle on fluid sensitive sequence. The lesion extended from the posterior column of the subarticular portion of the right acetabulum into the posterior superior right iliac bone subjacent to the right sacroiliac joint.

### 2.2. Pathological Findings

The initial biopsy revealed focal fibrosis and marrow that are slightly hypercellular with mild increase in plasma cells that are mostly scattered and comprise approximately 10% of the marrow cells. Immunohistochemical stains for CD 138, kappa, and lambda react positively, suggesting that they are polyclonal. Therefore, no clear evidence for plasma cell neoplasm is found. CD31 and CD34 immunostains marked for endothelial cells show focal vascular hyperplasia.

**Figure 3:**
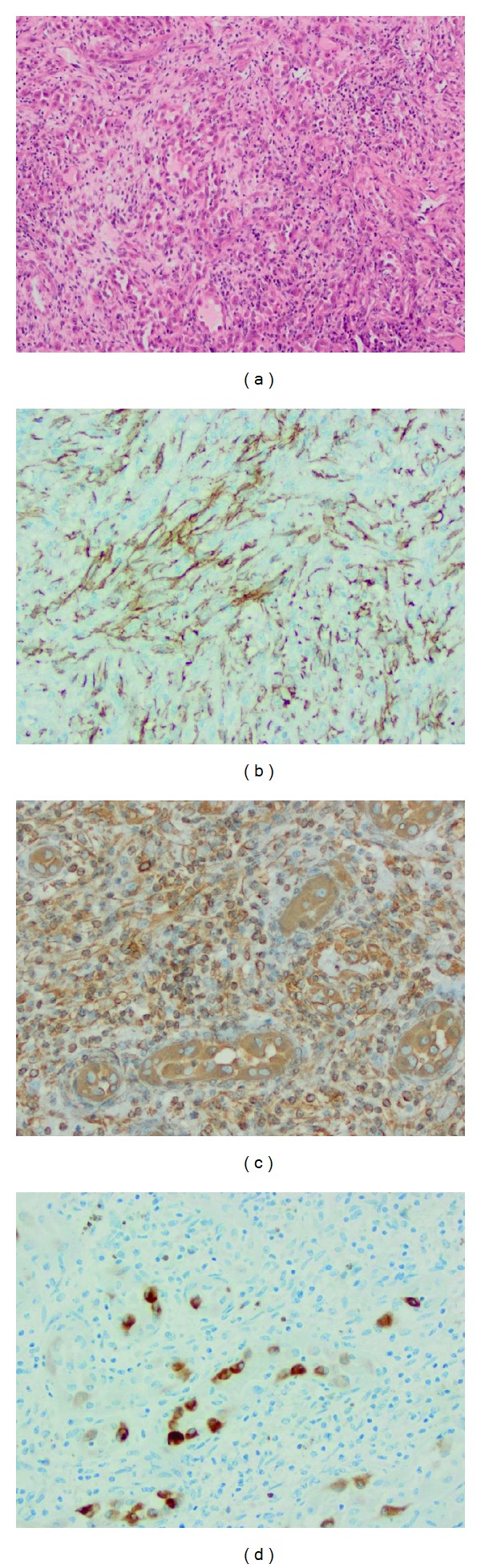
Histopathological characteristics of osseous epithelioid hemangioendothelioma of the iliac bone. Hematoxylin-eosin stain at 100x showing rounded to slightly spindled eosinophilic endothelial cells with nuclear atypia (a). Flattened cells that stain positively for anti-CD31 monoclonal antibody (b). Tumor cells stain nonspecifically for vimentin (c) and broad spectrum cytokeratin (d).

The curettage specimen consists of multiple fragments of soft tissue and bone measuring 5.5 × 5.5 × 0.8 cm in aggregate. Histological section reveals proliferation of well-formed thick walled vessels, anastomosing vascular channels lined by epithelioid cells in hyalinized and inflamed stroma. Neoplastic cells show moderate nuclear pleomorphism, abundant eosinophilic cytoplasm, and intracytoplasmic lumen formation (Figure 3(a)). Mitotic activity is rare with 2 mitoses per 10 high power fields. The tumor cells stain positively for vimentin, Factor VIII, CD31, and CD34 and faintly positive for broad spectrum cytokeratin and S100 protein (Figures 3(b)–3(d)). The specimen stains negatively for desmin, HHV8, ALK-1, and BCL2. Final pathologic diagnosis is atypical epithelioid hemangioendothelioma.

### 2.3. Radiation Therapy

**Figure 4:**
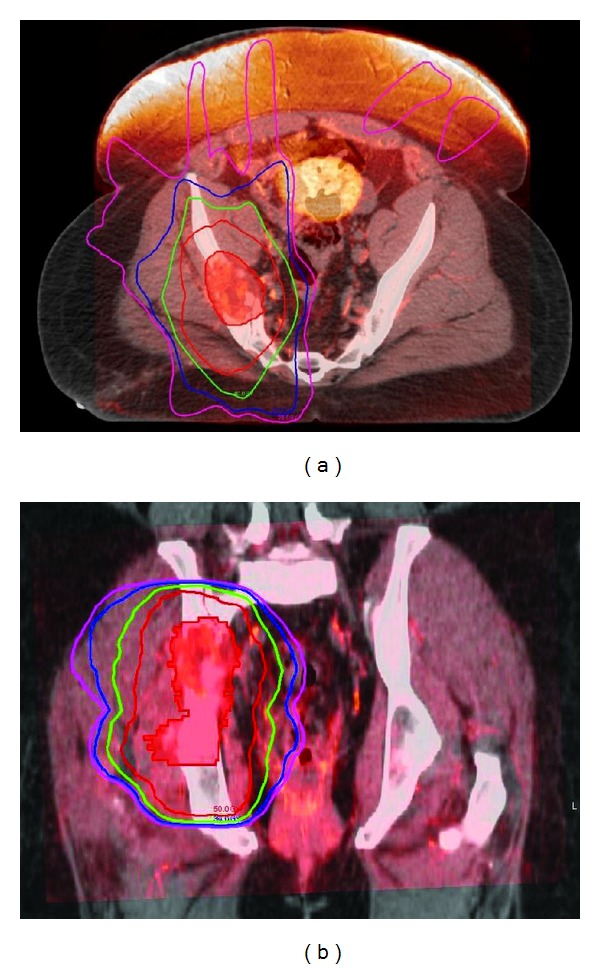
Fusion of the planning CT and the preoperative T1-weighted postcontrast MRI utilized to outline the clinical targeting volume (red colorwash) and to develop an Intensity Modulated Radiation Therapy plan depicting the isodose profiles for 50 Gray (red), 40 Gray (green), 30 Gray (blue), and 25 Gray (purple) in the axial (a) and the coronal (b) planes.

Given the extent of the disease involvement revealed at the time of curettage, an upfront complete resection with negative margins may result in morbidity and high probability of debilitation. Hence, it was the decision of the multidisciplinary tumor board that the patient undergoes upfront radiation therapy to reduce the tumor size and the extent of disease to facilitate surgical resection. She underwent CT simulation using vac loc bag to maintain daily treatment reproducibility prior to the plan generation. In order to reduce the potential dose to surrounding normal tissues such as ovary, bowel, bladder, rectum, and femoral heads, a five-field Intensity Modulated Radiation Therapy (IMRT) plan was utilized (Figure 4). MRI fusion to the planning CT was completed to outline the target volume. Radiation was delivered via 10 megavolt photon to a total dose of 5000 centigray (cGy) over 25 daily fractions at 200 cGy per fraction. Patient tolerated the entire radiation course with Radiation Therapy Oncology Group (RTOG) classification of grade one lower gastrointestinal toxicity, which consisted of mild loose stool, and grade two skin toxicity, which consisted of moist desquamation. The tumor experienced partial response to IMRT. Patient continued to exhibit biopsy proven disease three months after completion of radiation therapy. She underwent wide resection of right hemipelvis, cadaveric allograft reconstruction of right hemipelvis with open reduction internal fixation, and total right hip arthroplasty. The postoperative pathological specimen was consistent with atypical epithelioid hemangioendothelioma. All surgical margins were negative.

At approximately one year after completion of therapies, the patient remains free of malignancy.

## 3. Discussion

Epithelioid hemangioendothelioma falls under the broad histological category that also includes papillary intralymphatic angioendothelioma, spindle cell, retiform, and composite hemangioendothelioma. Historically, EH has shown similarities to histiocytoid hemangioma, cellular hemangioma, low grade anaplastic sarcoma, and angioendothelioma. EH has been correlated with translocation of (1; 3) (p36; q35) and (10; 14) (p13; q42) as well as deletions in chromosomes 11 and 12 [3].

EH was first described by Weiss and Enzinger in 1982 [4]. Since then, the authors published one of the largest series, consisting of 46 case reports, that revealed a local control of 87 percent, regional nodal control rate of 69 percent, and overall survival of 87 percent at two-year followup [5, 6]. EH involving the ilium accounts for less than five percent of osseous EH [7, 8].

Majority of EH patients present in the second and third decade of life with nonspecific signs and symptoms such as vague pain, edema, and vascular and neurological symptoms secondary to mass effect depending on the location of the tumor. There is no difference in incidence between male and female. Pathological fracture can occur in approximately ten percent of osseous EH [7, 8].

Workup includes imaging studies, and diagnosis is confirmed by pathological findings. On plain X-ray, osseous EH often presents as an expansive, well-demarcated, lytic mass [9, 10]. Ultrasonography may be utilized to detect vascularization as to differentiate from other highly vascular mass. Joint invasion, as in the present case, can be seen as a homogenous enhancement on contrast enhanced CT and MRI. EH, similar to other tumors of vascular origin, exhibits higher intensities than muscles but lower than fat on T1-weighted contrast MRI [7]. However, on T2-weighted MRI, osseous EH exhibits higher intensity than muscle and fat [8].

Gross pathological examination may show a tan, soft, and lobulated mass with scalloped borders. Microscopically, osseous EH exhibits network of irregular vascular channels lined by endothelial cells with high degree of anaplasia [11, 12], often embedded in chondroid-like or hyalinized stroma [8]. Osteoclastic giant cells, high mitotic activity, spindling of the neoplastic cells, and necrosis have been associated with aggressiveness of the tumor [13]. As in the present case, immunohistochemical analysis is positive for vimentin, Factor VIII, CD31, CD 34, cytokeratin, and S100 protein [8].

Outcome is most favorable for low grade lesions when complete excision can be achieved. However, treatments range from simple curettage to en bloc resection to radical resection of the tumor and surrounding structures or organs [14]. In the past, radiation has been utilized to treat unresectable tumors, as well as in the adjuvant setting for resectable or partially resectable tumors [8, 14]. Though a recommended definitive radiation dose for osseous EH has not been established by the National Comprehensive Cancer Network (NCCN), one can extrapolate the data from angiosarcoma, which have been treated with RT dose ranging from 4140 cGy to 6600 cGy in the definitive setting at 180–200 cGy per fraction [15–17]. Unlike fast dividing squamous cell carcinoma, small cell carcinoma, or adenocarcinoma, endothelioma and sarcoma are less responsive to radiation therapy. This difference in radiosensitivity is mainly secondary to differences in cell loss factor (*φ*) which represents the ratio of the rate of cell loss to the rate of new cell production or proliferation as exhibited by the equation *φ* = 1 − *T*_pot_/*T*_*d*_, where *T*_pot_ is the potential tumor doubling time and *T*_*d*_ is the actual tumor doubling time [18]. The cell loss factor for sarcoma has been measured to range from 10 to 55% [19–21]. On the other hand, carcinoma cell loss factor ranges from 70 to 93% [22, 23]. The pattern of cell loss is intimately related to apoptosis as a mode of cell death. In comparison to sarcoma, apoptosis plays a more predominant role in carcinoma cell population. Hence, fractionated radiation therapy, which causes *G*_2_/*M* cell cycle arrest in preparation for mitotic catastrophe and apoptosis, results in greater percentage of cell kill in carcinoma than sarcoma or endothelioma [18]. As a result, using radiation therapy as a sole modality for osseous EH requires a high dose and not as effective as complete surgical resection with negative margins. Such high dose requirements are difficult to achieve without spillage dose that affect adjacent normal tissues and organs resulting in radiation induced toxicity. Similar to limitations to achieving complete surgical resection, ability to administer adequate radiation dose is limited by the location of the primary tumor. In the current case, radiation therapy is used in the neoadjuvant setting with the intention of reducing the tumor size and the extent of the disease to facilitate complete surgical resection. The radiation dose is limited secondary to nearby structures such as the femoral head, pelvic bone, small bowel, large bowel, bladder, and rectum. Despite the utilization of IMRT to limit the spillage dose to adjacent normal tissues, unacceptable toxicity, such as femoral fracture, anemia, severe diarrhea, bowel perforation, rectal fistula, bladder irritation, hematuria, bladder perforation, and vaginal fibrosis, may ensue should higher dose of RT be administered.

## 4. Conclusion

Osseous EH involving the femur and ilium is rare and can be difficult to diagnose from the clinical and histopathological perspective. Depending on the anatomical location of the primary tumor, local control and cure may be difficult to achieve with a single modality of therapy. As such, a multidisciplinary team of radiologists, pathologists, surgeons, medical oncologists, and radiation oncologists are required to generate a plan of care that produce the highest chance for cure and the lowest morbidity. Given the rarity of this disease, further studies are needed to establish guidelines and elucidate the best sequence of therapeutic interventions. This case highlights a multidisciplinary collaborative approach to the management of osseous EH in a difficult anatomical location to be adequately addressed by a single modality. In this case the patient is able to achieve cure and local control.

## References

[1] Weiss SW, Goldblum JR, Enzinger FM, Weiss SW Hemangioendothelioma: vascular tumors of intermediate malignancy. *Soft Tissue Tumors*.

[2] http://www.bonetumor.org/unknown-tumor-soft-tissue/epithelioid-hemangioendothelioma.

[3] Evans HL, Raymond AK, Ayala AG (2003). Vascular tumors of bone: a study of 17 cases other than ordinary hemangioma, with an evaluation of the relationship of hemangioendothelioma of bone to epithelioid hemangioma, epithelioid hemangioendothelioma, and high-grade angiosarcoma. *Human Pathology*.

[4] Weiss SW, Enzinger FM (1982). Epitheloid hemangioendothelioma. A vascular tumor often mistaken for a carcinoma. *Cancer*.

[5] Gherman CD, Fodor D (2011). Epithelioid hemangioendothelioma of the forearm with radius involvement. Case report. *Diagnostic Pathology*.

[6] Weiss SW, Ishak KG, Dail DH, Sweet DE, Enzinger FM (1986). Epithelioid hemangioendothelioma and related lesions. *Seminars in Diagnostic Pathology*.

[7] Boutin RD, Spaeth HJ, Mangalik A, Sell JJ (1996). Epithelioid hemangioendothelioma of bone. *Skeletal Radiology*.

[8] Themistocleous GS, Papagelopoulos PJ, Petraki KD, Stillanessi EV, Partsinevelos AA, Sapkas GS (2005). A 23-year-old woman with complete paraplegia and anesthesia below the T8 level. *Clinical Orthopaedics and Related Research*.

[9] Ibarra RA, Kesava P, Hallet KK, Bogaev C (2001). Hemangioendothelioma of the temporal bone with radiologic findings resembling hemangioma. *American Journal of Neuroradiology*.

[10] Thananopavarn P, Smith JK, Castillo M (2003). MRI of angiosarcoma of the calvaria. *American Journal of Roentgenology*.

[11] Lezama-del Valle P, Gerald WL, Tsai J, Meyers P, La Quaglia MP (1998). Malignant vascular tumors in young patients. *Cancer*.

[12] Scholsem M, Raket D, Flandroy P, Sciot R, Deprez M (2005). Primary temporal bone angiosarcoma: a case report. *Journal of Neuro-Oncology*.

[13] Tsuji M, Ozaki T, Tsutsumi A (2002). Epithelioid hemangioendothelioma with osteoclast-like giant cells. *Pathology Research and Practice*.

[14] Aquilina K, Lim C, Kamel MH, Marks CJ, O’Sullivan MG, Keohane C (2005). Epithelioid hemangioendothelioma of the spine. Report of two cases. *Journal of Neurosurgery. Spine*.

[15] Hirsh AZ, Yan W, Wei L, Wernicke AG, Parashar B (2010). Unresectable retiform hemangioendothelioma treated with external beam radiation therapy and chemotherapy: a case report and review of the literature. *Sarcoma*.

[16] Mark RJ, Poen JC, Tran LM, Fu YS, Juillard GF (1996). Angiosarcoma: a report of 67 patients and a review of the literature. *Cancer*.

[17] Abraham JA, Hornicek FJ, Kaufman AM (2007). Treatment and outcome of 82 patients with angiosarcoma. *Annals of Surgical Oncology*.

[18] Hall EJ, Giaccia AJ (2012). *Radiobiology for the Radiologist*.

[19] Frankfurt OS (1967). Mitotic cycle and cell differentiation in squamous cell carcinomas. *International Journal of Cancer*.

[20] Frindel E, Malaise E, Tubiana M (1968). Cell proliferation kinetics in five human solid tumors. *Cancer*.

[21] Frindel E (1969). Proliferation kinetics of an experimental ascites tumor of the mouse. *Cell and Tissue Kinetics*.

[22] Reiskin AB, Berry RJ (1968). Cell proliferation and carcinogenesis in the hamster cheek pouch. *Cancer Research*.

[23] Reiskin AB, Mendelsohn ML (1964). A comparison of the cell cycle in induced carcinomas and their normal. *Cancer Research*.

